# *Callosobruchus* embryo struggle to guarantee progeny production

**DOI:** 10.1038/s41598-020-70178-9

**Published:** 2020-08-06

**Authors:** Azam Amiri, Ali R. Bandani

**Affiliations:** 1 College of Geography and Environmental Planning, University of Sistan and Baluchestan, Zahedan, Iran; 2 Department of Plant Protection, College of Agriculture and Natural Resources, University of Tehran, Tehran, Iran

**Keywords:** Developmental biology, Ecology, Evolution, Zoology

## Abstract

We conducted a series of experiments to test insect embryo capability to survive and increase reproductive investment during early development after short exposure to essential oils. We used *Callosobruchus maculatus* as a model insect and eucalyptus leaf and flower essential oils. Both essential oils exhibited toxicity against *C. maculatus* embryos and adults. However, flower essential oil was more toxic. A fetus exposed to essential oils tried to make the best of a bad situation and compensate essential oils harmful effects in the later life stages. Insect progeny production guarantee resulted in a trade-off between reproduction and female longevity. The insect also could alter fitness and reproductive behavior including, mating latency reduction, copulation duration increase, and copulation success rate raise in adulthood. Flower essential oil-exposed embryos were more successful in increasing copulation duration, and leaf essential oil-exposed embryos achieved more copulation success and less mating latency. These consequences persisted until F1 generation that was not directly exposed to essential oil. However, the F2 generation could concur with the harmful effects of essential oils. *C. maculatus* embryo might use epigenetic mechanisms to guarantee progeny production. Reproductive behavior changes and the trade-off can be evolutionary mechanisms to save species from possible extinction in deleterious situations.

## Introduction

Embryo exposure to some substances can have profound impacts on the life history strategies of many vertebrates lead to differences in adult competitive abilities and alternative reproductive tactics that possess evolutionary importance^1^.

Insects, with 450 million years history of living on earth, are the most successful life and useful models for the research of invertebrate animal features. Terrestriality, fight, complete metamorphosis, and eusociality have mentioned as four major adaptive features of insects^2^. However, insects possess other evolutionary features worth studying, such as insect behavioral immunity against different environmental stressors^3^. Environmental stressors such as nutrient availability, toxin or pathogen exposure, can severely restrict the reproduction ability of an organism and cause parental attempts to fight against it^4^.

Plant essential oils have been used for arthropod pest control as promising attractive alternatives to many synthetic pesticides because of their fast degradability properties, safety to humans and environment, and especially in case of pesticide resistance developing. Essential oils as fumigants or contact insecticides influence insect physiology by disruption of primary metabolic pathways result in rapid death, longevity reduction, and alteration of oviposition. Plant volatiles could also cause behavioral responses in insects and synergize or increase insect responses to sex pheromones^5–10^.

Due to the potential use of essential oils as natural biocides, lethality or effects on development have been well studied. Non-lethal consequences, however, remain under-documented^11^.

Scientists have demonstrated environmental stimulus (such as a toxin or toxicant exposure) could lead to organism gene expression changes to overcome a new situation. Epigenetic is changes in gene expression by heritable modifications to the DNA molecule but not gene sequence base changes because of different environmental stressors. It can lead to heritable adaptation in natural populations^12^.

Epigenetic mechanisms can be exploited to alter gene expression. Immediate changes in gene expression are involved in not only the toxin metabolism but also critical biological processes. Some of the changes in gene expression are transient, and some epigenetic changes could be persistent and last after termination of exposure. Some even inherited in later generations that did not experience exposure directly. Epigenetic changes take place in hours, but the results could proceed for a lifetime^13,14^.

To the best of our knowledge, insect embryo ability to survive against exposure to plant essential oil and compensate the damages to product progeny has not well-discussed. In this study, we used the cowpea weevil, *Callosobruchus maculatus* (Coleoptera: Chrysomelidae), as a model insect and *Eucalyptus camaldulensis* (Myrtales: Myrtaceae) flower and leaf essential oils to study probable attempt and success of embryo to avoid reproduction failure in adulthood by improving behavioral fitness.

## Results and discussion

### Fumigant toxicity

The results demonstrated that eucalyptus, both leaf and flower essential oils were toxic to *C. maculatus* and exhibited intense insecticidal activity. However, flower essential oil was more toxic. Besides, fumigant toxicity varied with the type of plant part, oil concentration, and exposure time (Supplementary Tables 1 and 2).

Mortality of *C. maculatus* adults increased with increasing oil concentration and time of exposure. The flower essential oil showed a more robust fumigant activity. For example, LC_50_ at 24 h was 132.7 µl/l_air_ for the flower essential oil compared to 174.2 µl/l_air_ for the leaves (Supplementary Table 1).

**Figure 1:**
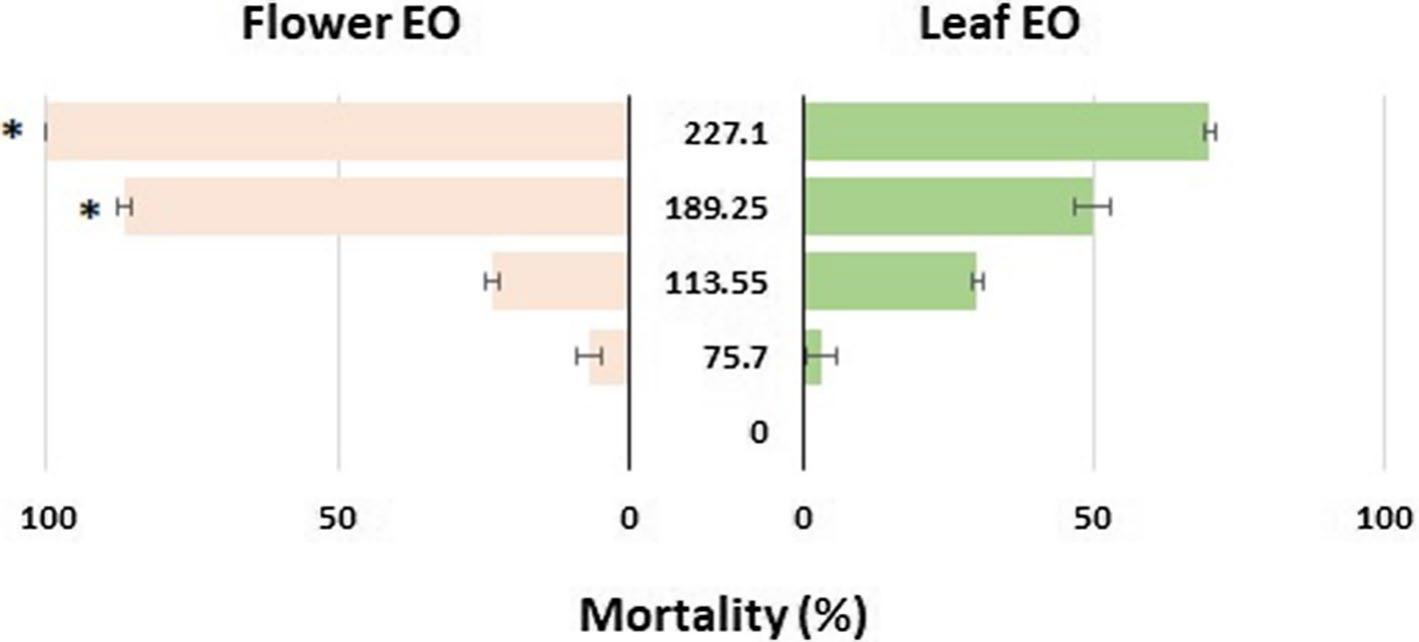
Mortality caused by different concentrations of eucalyptus leaf and flower essential oils (EO) in *C. maculatus* 24 h post-treatment. The asterisks indicate significant differences between leaf and flower essential oils (*P* < 0.05).

Mortality caused by two essential oils compared 24 h post-treatment. Two concentrations of 189.25 and 227.1 µl/l_air_ flower essential oil induced significantly more mortality (86.6 and 100%, respectively) than leaf essential oil (50 and 70%, respectively) in the same concentration (Fig. 1).

The effectiveness of essential oils depends on many factors, such as the chemical composition of essential oils, the mode of application and the post-application temperature of the environment, etc. For example, the toxicity of *Thymus vulgaris* essential oil and its major constituents against the larvae of *Culex quinquefasciatus* and *Spodoptera littoralis* were increased with rising temperature^15^.

Some studies have documented that sublethal exposure to plant essential oils, or environmental changes (e.g., host changes) could impact the fitness of stored product insect pests. For example, clove and cinnamon essential oils have reported as toxic as the pyrethroid-based insecticide deltamethrin against *C. maculatus* and severely decreased adult emergence and egg number^16^. Host shift effects from kidney beans to cranberry beans have been studied in the bean weevil *Acanthoscelides obtectus*, and reproductive performance has been evaluated after exposure to clove and cinnamon essential oils. The results indicated that clove essential oil was more effective when insects were reared on cranberry bean masses and caused higher mortality^17^.

With the highest concentration (227.1 µl/l_air_), the flower essential oil took the shortest time (LT50 = 4.4 h) to cause 50% mortality compared to leaves (LT50 = 9.6 h) (Supplementary Table 2).

**Figure 2:**
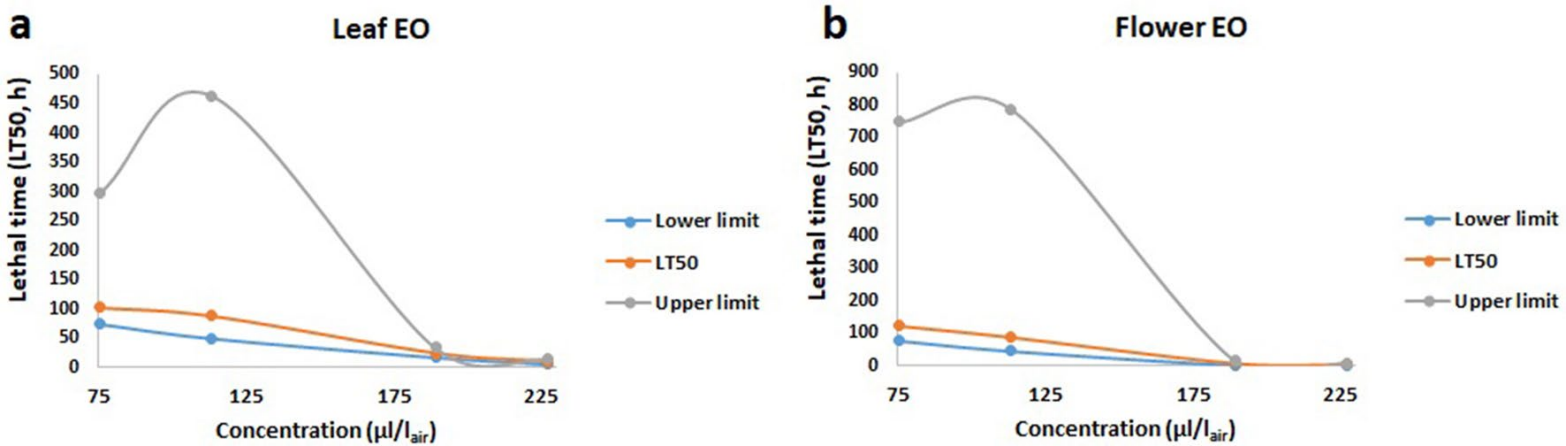
Median lethal time (LT_50_) at increasing concentrations of eucalyptus leaf (**a**) and flower (**b**) essential oils against *C. maculatus*. Increasing concentrations led to decreasing LT_50_.

Flower essential oil reached 100% mortality after 24 h at the highest concentration (227.1 µl/l_air_), while leaf essential oil caused 70% mortality at the same concentration and time point (Supplementary Fig. 1). Median lethal time (LT_50_) significantly decreased with the leaf and flower increased oil concentrations (Fig. 2).

### The trade-off between reproduction and longevity

**Figure 3:**
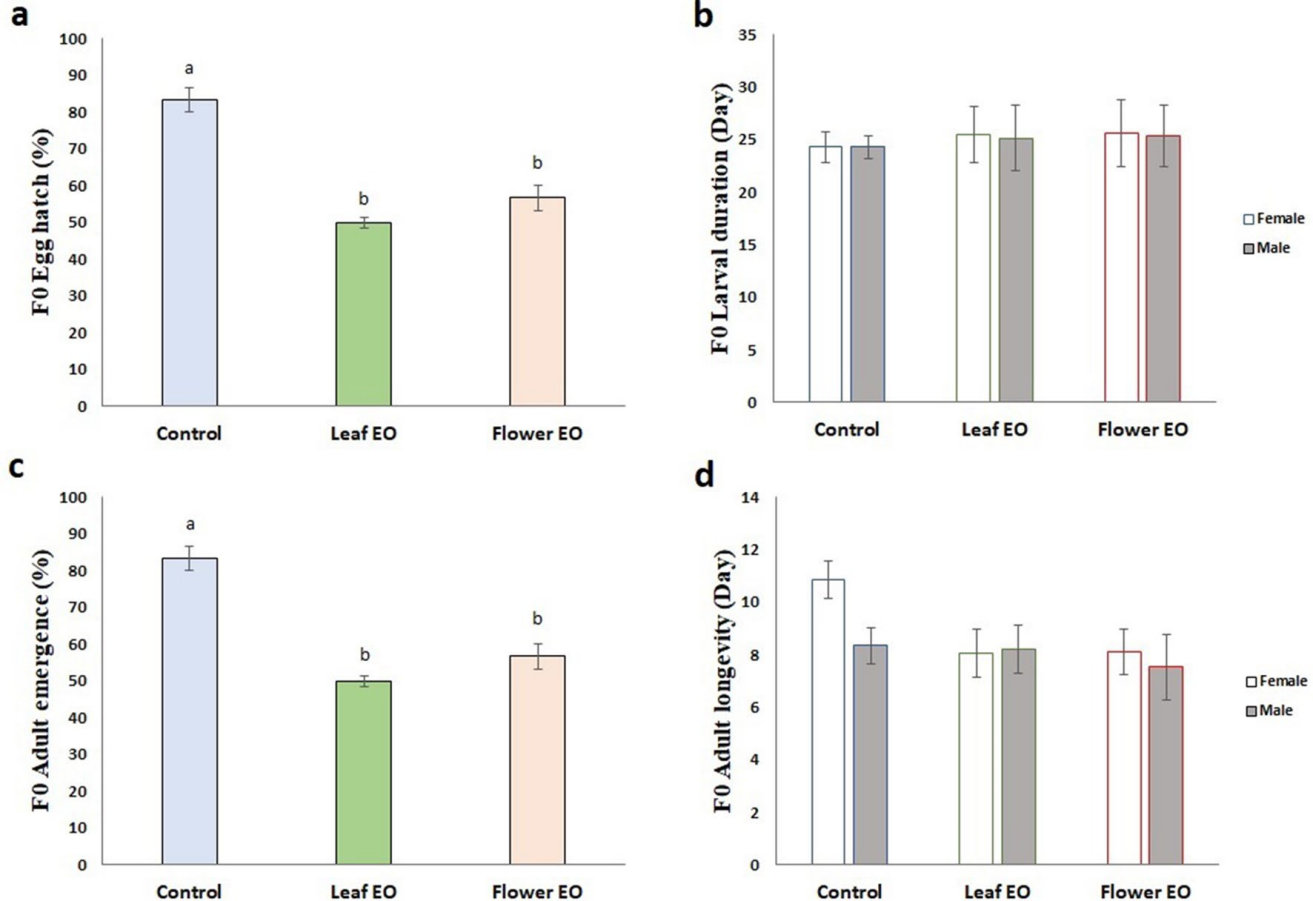
Effect of eucalyptus leaf and flower essential oils (EO) on different biology parameters of *C. maculatus* F0 generation. (**a**) F0 egg hatch (%); (**b**) F0 larval duration (day); (**c**) F0 adult emergence (%); (**d**) F0 adult longevity (day). Essential oils did not influence F0 larval duration and adult longevity. However, decreased F0 egg hatch (%) and adult emergence. Different letters indicate significant differences between the treatments (*P* < 0.05).

We observed that both essential oils were toxic to eggs of *C. maculatus* and significantly decreased hatch rate by about 50% (56.6 ± 3.3% for flower essential oil and 50 ± 1.5% for leaf essential oil). The control egg hatch rate was 83.33 ± 3.3% (Fig. 3a). Interestingly, survivors could compensate harmful effects of embryo exposure to essential oils, and hence, control and treatments had statistically the same larval duration (Fig. 3b). Insects have developed evolutionary strategies to compensate for unfavorable situations encountered during different life stages. For example, insects that experience a period of lack of food and nutrition grow faster than those who were in a normal situation to compensate for initial failure^18^.

However, a significantly remarkable reduction in adult emergence was observed in essential oil-treated insects (56.6 ± 3.3% for flower essential oil and 50 ± 1.5% for leaf essential oil) when compared to the control (83.33 ± 3.3%) (Fig. 3c). Adult emergence reduction was due to egg mortality because it was the same as egg hatch, and no larval mortality was recorded.

Embryo short time exposure to the essential oils influenced female adult longevity (Fig. 3d). The longevity was 10.8 days for control females and 8.0 and 8.1 days for leaf and flower essential oil-treated females, respectively. Treated and untreated adult males had statistically the same longevity. Male longevity was shorter than that of females (Fig. 3d).

**Figure 4:**
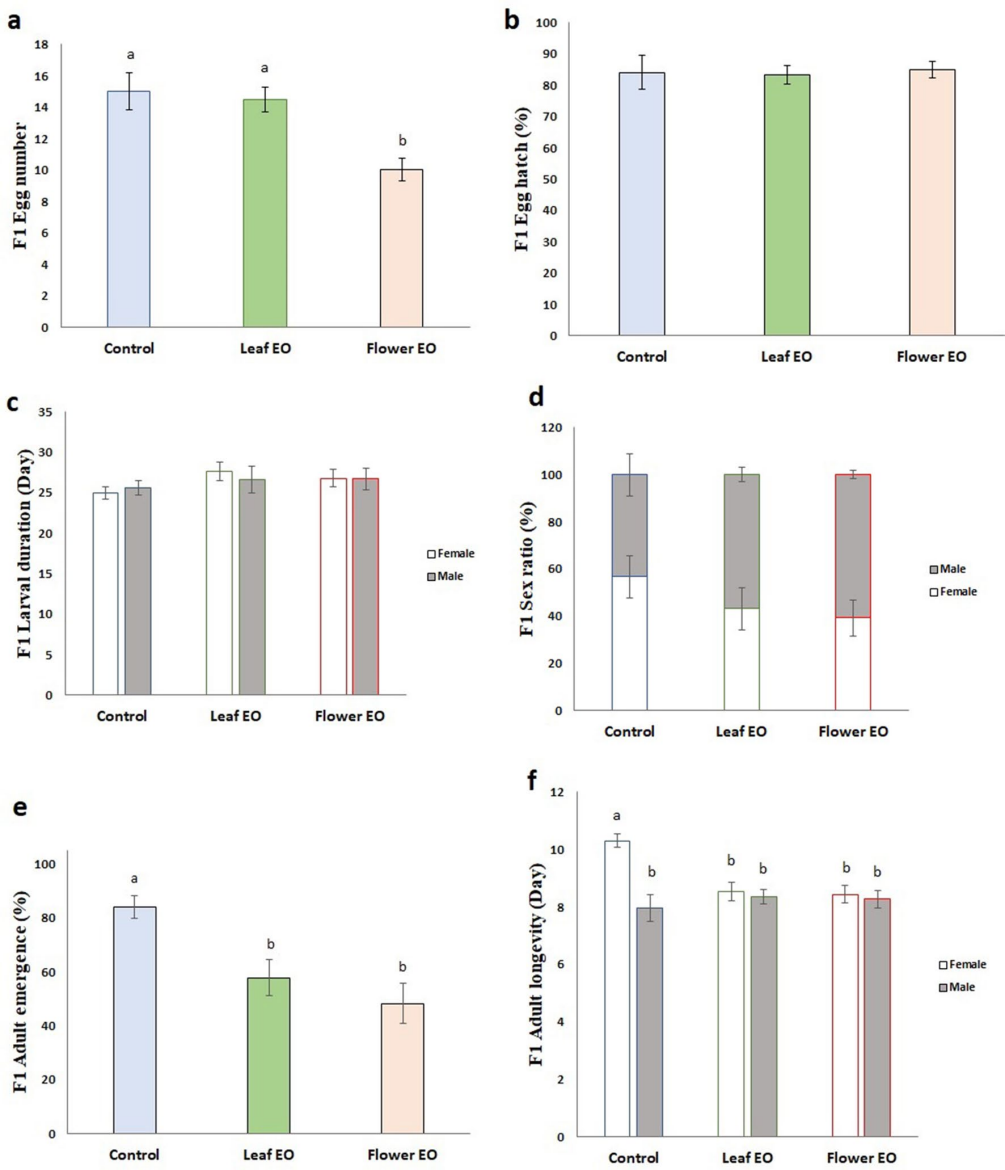
Effect of eucalyptus leaf and flower essential oils (EO) on different biology parameters of *C. maculatus* F1 generation. (**a**) F1 egg number; (**b**) F1 egg hath (%); (**c**) F1 larval duration (day); (**d**) F1 sex ratio; (**e**) F1 adult emergence (%); (**f**) F1 adult longevity (day). Flower essential oil treatment decreased F1 egg numbers, and both essential oils caused a decrease in F1 adult emergence and female longevity. Different letters indicate significant differences between the treatments (*P* < 0.05). Essential oils had no significant effects on F1 egg hatch (%), larval duration, and sex ratio.

It is worth noting that F0 embryo exposure to flower essential oil significantly reduced F1 egg number (10 ± 0.7) compared to control (15 ± 1.1) and leaf essential oil (14.4 ± 0.7) group (Fig. 4a). Therefore, there was a fitness consequence of essential oil stress by a trade-off between reproduction and female longevity in leaf essential oil-treated embryo. Thus, the improvement in reproduction was costly in terms of longevity decrease to avoid further reduction of egg numbers.

A trade-off occurs when an improvement in one life-history trait (improving fitness) is combined with a decrease in another life-history feature (reducing fitness). So that the fitness value is balanced against fitness costs by increasing one trait by decreasing another trait. Trade-offs are typically defined by negative genetic or phenotypic correlations between individual fitness components within a population^19^. In *Bemisia tabaci*, a trade-off between longevity and egg numbers has been reported^20^.

A large number of studies have demonstrated that sublethal concentrations of essential oils influence insect biology and inhibit insect oviposition by reduction of egg number or hatchability. For example, lemongrass, rosemary, *Vanillosmopsis arborea*, *Eucalyptus camaldulensis,* and *Heracleum persicum* essential oils caused egg number reduction in *C. maculatus*^10,21–23^. It is noteworthy that in the all mentioned experiments, sexually mature adults were exposed to essential oils. However, we used the embryo, and the effect of essential oil exposure persisted until adulthood and F1 generation.

F1 egg quality, larval duration, and sex ratio were not affected by F0 embryo exposure to essential oils; hence egg hatch rate was statistically equal in the treatments and control. Sex ratio was 1:1 (female/male) in control and treatments (Fig. 4b–d).

Although leaf essential oil-treated embryos attempted to deposit the same egg number, with the same hatch rate as control in adulthood, all larvae could not make it until adulthood, and F1 adult emergence rate was significantly lower than control (84 ± 4%) in both essential oils group offspring (57.9 ± 6% and 48.3 ± 7% for leaf and flower essential oils, respectively) (Fig. 4e).

Interestingly, F1 females that did not directly expose to essential oil fumigation exhibited significant reductions in their longevity like their parents (10.3, 8.5 and 8.4 days for control, leaf, and flower essential oils, respectively) (Fig. 4f).

Sublethal doses or short-term exposure of insects to essential oils have been found to affect insect fertility, vitality, and longevity, even in the F1 generation. *Carlina acaulis* root essential oil topical application reduced female *Musca domestica* longevity and egg number. Mortality of F1 larvae and pupae were increased, and F1 adult emergence were decreased^24,25^. Besides, *M. domestica* adult exposure to thyme oil sublethal doses negatively impacted adult longevity, F1 vitality, and F1 fecundity^26^.

The maize weevil, *Sitophilus zeamais* sublethal exposure to clove and cinnamon essential oils caused similar insecticidal toxicity and reduced respiratory rate and parent longevity and influenced progeny fitness by accelerating offspring emergence and producing heavier progeny^27,28^.

We recorded the F2 generation egg number and hatch rate to realize if the effects of short-time exposure of the embryo to essential oil could be passed on to future generations after the F1 generation. The F2 generation could completely concur with the deleterious effects of essential oils, and; thus, egg numbers and hatching rates were statistically the same in treatments and control (Supplementary Fig. 3).

### Reproductive behavior fitness

The environment can affect reproductive behavior in males and females, including mate recognition, courtship, mating, and post-mating behavioral changes. Mating behavior in insects is a significant reproductive process^29^.

**Figure 5:**
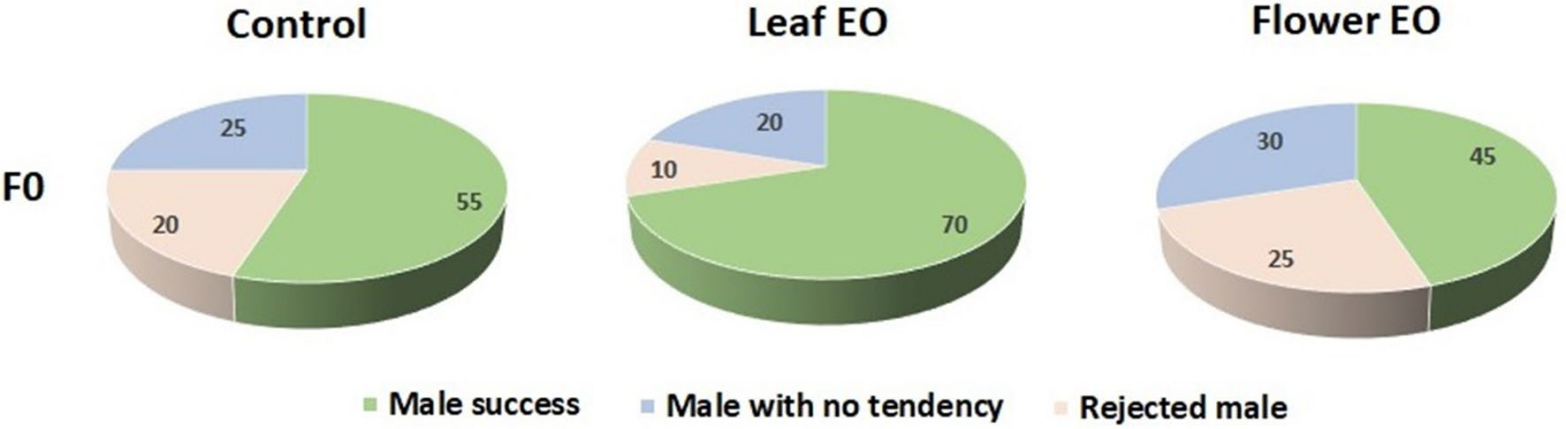
Effect of eucalyptus leaf and flower essential oils (EO) on male copulation parameters of *C. maculatus* F0 generation.

We demonstrated that leaf essential oil-exposed male embryos improved their reproductive behavior in adulthood and achieved more copulation success (70%) and less mating latency (85.9 s) compared to control (55% and 149.3 s) and flower essential oil group (45% and 120.8 s) (Fig. 5). Some studies have documented that exposure to the aroma of essential oil or particular plant compounds increases male insect mating competitiveness^30–32^. For example, ginger root oil, tea tree oil, and orange oil are involved in male competitiveness of the Mediterranean fruit fly, *Ceratitis capitata*^32,33^, and grapefruit oil enhanced *Anastrepha ludens* male mating success^34^. A single Plant volatile exposure increased the mating tendency of both sexes in the adult olive fruit flies *Bactrocera oleae*^35^.

**Figure 6:**
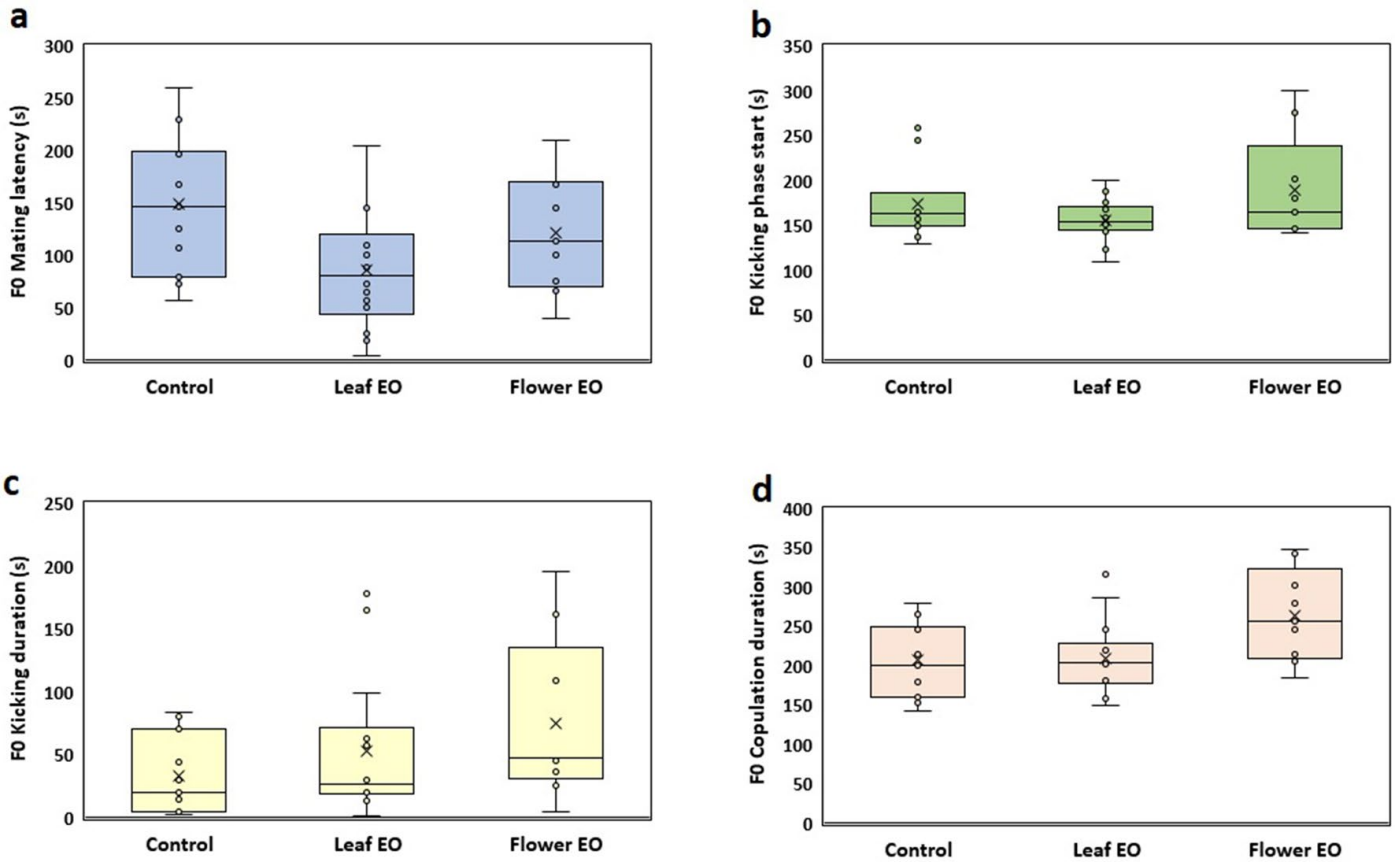
Effect of eucalyptus leaf and flower essential oils (EO) on different copulation parameters of *C. maculatus* F0 generation. (**a**) F0 mating latency (s); (**b**) F0 kicking phase start; (**c**) F0 kicking duration (s); (**d**) F0 copulation duration (s). Both essential oils significantly decreased male mating latency, and flower essential oil significantly increased copulation duration (*P* < 0.05). Essential oils did not impact female kicking phase start and duration.

Our results showed that flower essential oil-exposed embryos struggled to fight against future deleterious effects of essential oil on their potential reproductive success and finally were significantly more successful in increasing copulation duration time (264.4 s) compared to control (207.1) and leaf essential oil group (209.3) (Fig. 6d). *C. maculatus* females prefer short copulations due to physical injuries to their reproductive tracts^36^, whereas it was shown that in insects, the duration of copulation increases the reproductive success of males^37^. Some studies have shown that copulation behavior and duration can directly affect insect reproductive fitness and ability. Prolonged copulations increased male fertilization success in the damselfly, *Ceriagrion tenellum*, and two aphidophagous ladybirds^38,39^. Based on another study^40^, prolonged copulations not only did not hurt *C. maculatus* females but also could increase lifetime fecundity and material benefit that females derive from the increased ejaculate size. Females with long copulation duration deposited more eggs than females with shorter copulations.

Reproductive behavioral compensation mechanisms have been reported in the butterfly *Pararge aegeria*. Males that experienced diet with low nutritional quality during larval development could not monopolize an energetically costly territory similar to well-provided males. However, they compensated this weakness with a patrolling tactic to maximize reproductive success^41^. In insects and other animals, lower-quality individuals that influenced by environmental conditions try to make the best of an unfavorable situation and maximize their reproductive success by adopting alternative tactics^42^.

We did not observe significant differences in further examined reproductive behaviors among control and treated groups including, the number of males with no tendency to copulation, number of male with the struggle to start mating but rejected by females, and kicking phase duration of females to terminate copulations (Fig. 6).

**Figure 7:**
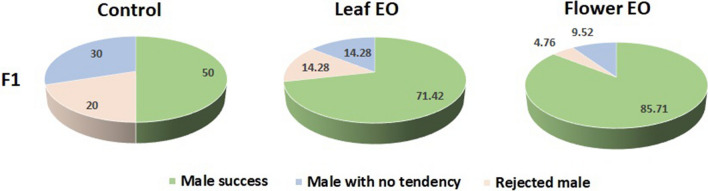
Effect of eucalyptus leaf and flower essential oils (EO) on male copulation parameters of *C. maculatus* F1 generation.

**Figure 8:**
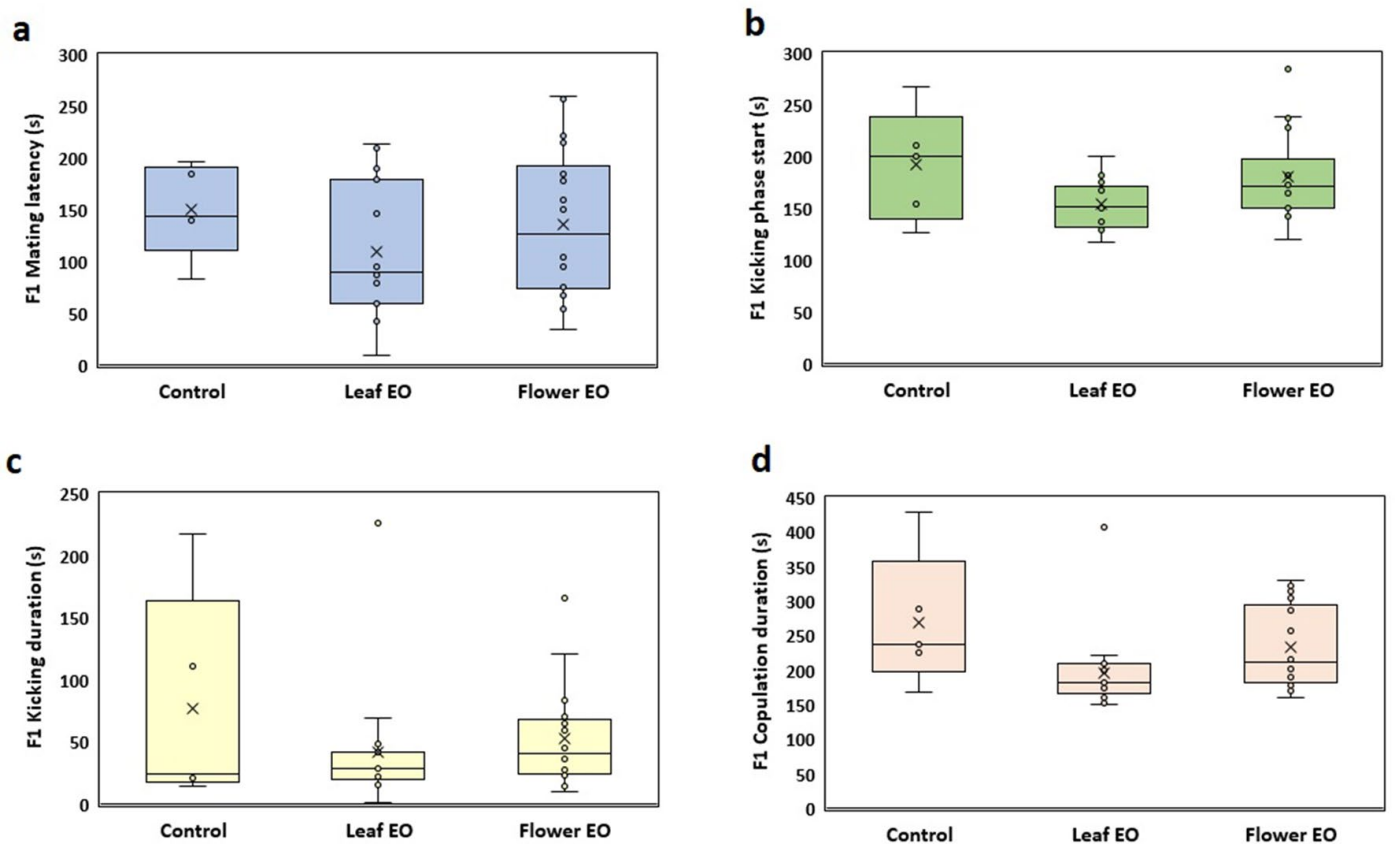
Effect of eucalyptus leaf and flower essential oils (EO) on different copulation parameters of *C. maculatus* F0 generation. (**a**) F0 mating latency (s); (**b**) F0 kicking phase start; (**c**) F0 kicking duration (s); (**d**) F0 copulation duration (s). There was no significant difference in the evaluated parameters (*P* > 0.05).

In F1 generation, both essential oil groups were still affected by their parents early-life exposure to essential oils; hence, both improved fitness by reproductive behavioral changes and male copulation success rate increased (85.7% and 71.42% for flower and leaf essential oils, respectively) in contrast to control (50%) (Fig. 7). There was no significant difference in other reproductive behaviors in the F1 generation (Fig. 8).

External stresses such as exposure to some substances or chemical pollutants can indeed have long-lasting effects on metabolism, development, and gene expression, sometimes even in subsequent generations^43^. Exposure to different volatile plant compounds can have long-term consequences on insect physiology as well as evolutionary adaptation^44^.

Epigenetic changes during early embryonic development will be amplified during development by cell division, and therefore influence a high amount of cells in the fully grown organism. However, when epigenetic changes arise in adult cells, they remain limited to those cells or a specific tissue^45^.

## Conclusion

In conclusion, our study demonstrates that *C. maculatus* embryo, after exposure to an environmental stressor like essential oils, struggles to compensate deleterious effects even by changing reproductive behavior to increase fitness and guarantee progeny production or trade-off between longevity and fecundity. Epigenetic is the mechanism that can transfer gene expression alteration template during different life stages and even to the later generations. It can be an evolutionary mechanism to save the species from possible extinction in deleterious environmental situations.

Also, our findings showed that botanical insecticides like eucalyptus leaf and flower essential oils could be used in *C. maculatus* control programs, especially in warehouses, as a substitute for conventional pesticides after further investigation.

## Materials and methods

### Insect colony

The strain of the cowpea weevil, *C. maculatus*, was maintained on black-eyed peas, *Vigna unguiculata* under laboratory conditions of 30 ± 1 °C and 50 ± 5% RH under 16L: 8D photoperiod. All experiments were accomplished under the same conditions. The newly emerged (< 24 h-old) adults and eggs (< 24 h-old) were chosen to set up the experiments.

### Plant materials

Flowers and leaves of *Eucalyptus camaldulensis* were collected from Zahedan, Sistan and Baluchestan province, Iran (Latitude: 29.4519, Longitude: 60.8842 and Elevation above sea level: 1,352 m). The plant samples were air-dried at room temperature for one week and then were hydrodistilled to extract the essential oils.

### Essential oil extraction

Dried flowers and leaves of *E. camaldulensis* (300 g) were grounded, and then essential oils were extracted by hydrodistillation in a Clevenger apparatus for 3 h. After extraction, water was removed by anhydrous sodium sulfate, and the extracted oil was stored in a dark box in a refrigerator at 4 °C.

### Fumigation bioassay

We used the newly emerged (< 24 h-old) adults of *C. maculatus* to set up the fumigation bioassay. We deposited each ten freshly emerged adults in a Petri dish (diameter 60 mm), which its top covered with filter paper. Based on an initial dose-setting experiment, 2, 3, 5, and 6 µl of *E. camaldulensis* leaf and flower essential oils (corresponding to essential oil concentrations of 75.7, 113.55, 189.25 and 227.1 µl/l_air_) were applied to the filter paper pieces. We used the same concentrations for both leaf and flower essential oils to be able to compare different effects of essential oils. Then, the Petri dishes' edges were sealed with parafilm. Each concentration and control (without essential oil) was replicated four times. Mortality was recorded at 6, 12, 24, 48, and 72 h after treatments. Insects with no movement of leg or antenna were considered as dead^36^.

### The embryo exposure to essential oils and insect biological parameters

Based on the bioassay, the embryo exposure to the concentration of 75.7 µl/l_air_ caused almost 50% of hatching in both essential oils (Supplementary Fig. 2). Therefore, 150 eggs were fumigated with leaf essential oil and 150 eggs with flower essential oil for 24 h. Also, 75 eggs that achieved no treatment considered as control. Treated eggs were placed in Petri dishes individually. Total numbers of eggs hatched were counted after 7 days. The daily observation was done, and F0 larval duration, adult emergence, and longevity were monitored every day. Since adults emerged, males and females were paired and checked daily to record their survival and the numbers of laid eggs. The experiments continued until all of the individuals died. Insects were allowed to oviposit 24 h to obtain the F1 egg number and hatch. Then seeds with eggs were transferred to a separate Petri dish. The experiment was repeated three times, and F1 adult emergence and longevity were recorded daily. Egg number and hatch rate were documented for F2 generation, too.

### Copulation test

We collected virgin males and females by removing adults as they emerged. We used 120 newly emerged (< 24 h-old) virgin adults to do copulation test. The male was placed in a Petri dish, and then immediately, a female was transferred. Pairs were given 5 min to mate. The pair's behavioral changes were monitored, and mating latency, the start of copulation, the start of a male kicking by female, and termination of copulation were recorded. If a male does nothing during this time, it was recorded as male without tendency. If a male tried to start copulation and female actively rejected males and prevent copulation, it was recorded as male rejection.

### Statistical analysis

One-way ANOVA analysis was performed using SPSS version 26.0 to compare egg number, egg hatch rate, larval duration, and adult longevity followed by Tukey’s test for mean separation. Statistical significance was established as *P* < 0.05. All other comparisons between treatments were analyzed using student’s t-test at 5% level. The LC_10_, LC_50_ and LC_90_, LT_10_, LT_50_, and LT_90_, as well as their respective 95% confidence intervals, were calculated by probit analysis (Polo Plus software).

## Supplementary information

Supplementary information 1.

Supplementary information 2.
