# Supplementary material for: Callosobruchus embryo struggle to guarantee progeny production
Source: Sci Rep. 2020 Aug 6;10:13269. doi: 10.1038/s41598-020-70178-9 (PMC7413255; doi:10.1038/s41598-020-70178-9)
Supplement: Supplementary file 1 — Supplementary information 1. [file 41598_2020_70178_MOESM1_ESM.docx]

***Callosobruchus* embryo struggle to guarantee progeny production**

Azam Amiri^*1^ and Ali R. Bandani^2^

1. College of Geography and Environmental Planning. University of Sistan and Baluchestan, Zahedan, Iran.

2. Department of Plant Protection, College of Agriculture and Natural Resources, University of Tehran, Iran.

^*^Corresponding author email: azamamiri@eco.usb.ac.ir

Supplementary Table 1. The lethal concentrations at different exposure times to eucalyptus leaf and flower essential oils against *C. maculatus*.

| Plant part | Time (h) | n | Slope±SE | χ2 | LC_10_ (µl/l_air_) | LC_50_ (µl/l_air_) | LC_90_ (µl/l_air_) | P |
| --- | --- | --- | --- | --- | --- | --- | --- | --- |
| Leaf | 6 | 270 | 5.62±1.70 | 5.69 | 157.7 (108.7-181.4) | 266.6 (227.0-439.7) | 450.6 (324.2-1558.2) | 0.002 |
| Leaf | 12 | 270 | 3.81±0.85 | 5.44 | 99.6 (64.1-122.1) | 216.1 (183.2-291.0) | 468.7 (331.2-1095.6) | <0.0001 |
| Leaf | 24 | 270 | 4.13±0.78 | 3.16 | 85.3 (57.6-104.5) | 174.2 (151.3-208.1) | 355.8 (275.7-597.1) | <0.0001 |
| Leaf | 48 | 270 | 3.70±0.69 | 11.34 | 61.5 (30.8-82.6) | 136.7 (110.9-167.2) | 303.3 (227.7-592.2) | 0.006 |
| Leaf | 72 | 270 | 4.21±0.72 | 7.04 | 58.3 (37.4-73.8) | 117.5 (99.5-135.0) | 236.8 (195.0-334.9) | <0.0001 |
| Flower | 6 | 270 | 4.75±1.08 | 5.84 | 117.8 (83.6-139.2) | 219.1 (188.1-291.1) | 407.4 (302.2-852.3) | <0.0001 |
| Flower | 12 | 270 | 6.87±1.10 | 6.76 | 103.7 (82.7-118.8) | 159.4 (143.4-177.7) | 244.9 (213.1-310.0) | <0.0001 |
| Flower | 24 | 270 | 7.52±1.06 | 7.49 | 89.7 (74.3-101.4) | 132.7 (119.9-146.9) | 196.4 (173.7-237.1) | <0.0001 |
| Flower | 48 | 270 | 6.20±0.93 | 4.91 | 74.5 (58.3-86.6) | 119.9 (106.6-134.0) | 192.8 (167.4-242.0) | <0.0001 |
| Flower | 72 | 270 | 5.95±0.93 | 10.48 | 68.2 (46.9-82.6) | 112.0 (95.5-129.6) | 183.9 (154.0-257.0) | <0.0001 |

Supplementary Table 2. The lethal times at different exposure concentrations of eucalyptus leaf and flower essential oils against *C. maculatus*.

| Plant part | Concentration (µl/l_air_) | Slope±SE | χ2 | LT_10_ (h) | LT_50_ (h) | LT_90_ (h) | P |
| --- | --- | --- | --- | --- | --- | --- | --- |
| Leaf | 75.7 | 2.88±0.85 | 9.44 | 37.06 (20.7 -47.9) | 103.2 (73.8 - 298.2) | 287.5 (147.2 - 3315.1) | 0.001 |
| Leaf | 113.55 | 1.07±0.30 | 4.40 | 5.6 (0.6-11.2) | 88.6 (49.6-462.6) | 217.4 (91.5-325.6) | 0.002 |
| Leaf | 189.25 | 1.64±0.29 | 7.09 | 4.01 (1.4-6.8) | 24.2 (17.5-33.6) | 146.4 (84.7-429.5) | <0.0001 |
| Leaf | 227.1 | 1.66±0.31 | 1.98 | 1.6 (0.3-3.3) | 9.6 (5.7-13.5) | 57.2 (37.6-126.2) | 0.001 |
| Flower | 75.7 | 2.43±0.78 | 3.01 | 36.5 (20.1 -51.2) | 122.6 (76.6 - 749.0) | 411.48 (167.8 - 19060) | 0.002 |
| Flower | 113.55 | 1.00±0.31 | 1.51 | 4.7 (0.3-10.0) | 87.9 (45.9-787.6) | 1640.9 (314.5-0.13266E) | 0.004 |
| Flower | 189.25 | 1.58±0.33 | 17.02 | 1.4 (0.0-3.6) | 9.0 (3.3-14.2) | 58.0 (33.0-275.3) | 0.001 |
| Flower | 227.1 | 1.95±0.44 | 18.27 | 0.9 (0.0-2.7) | 4.4 (0.7-7.7) | 20.1 (12.6-56.2) | 0.018 |


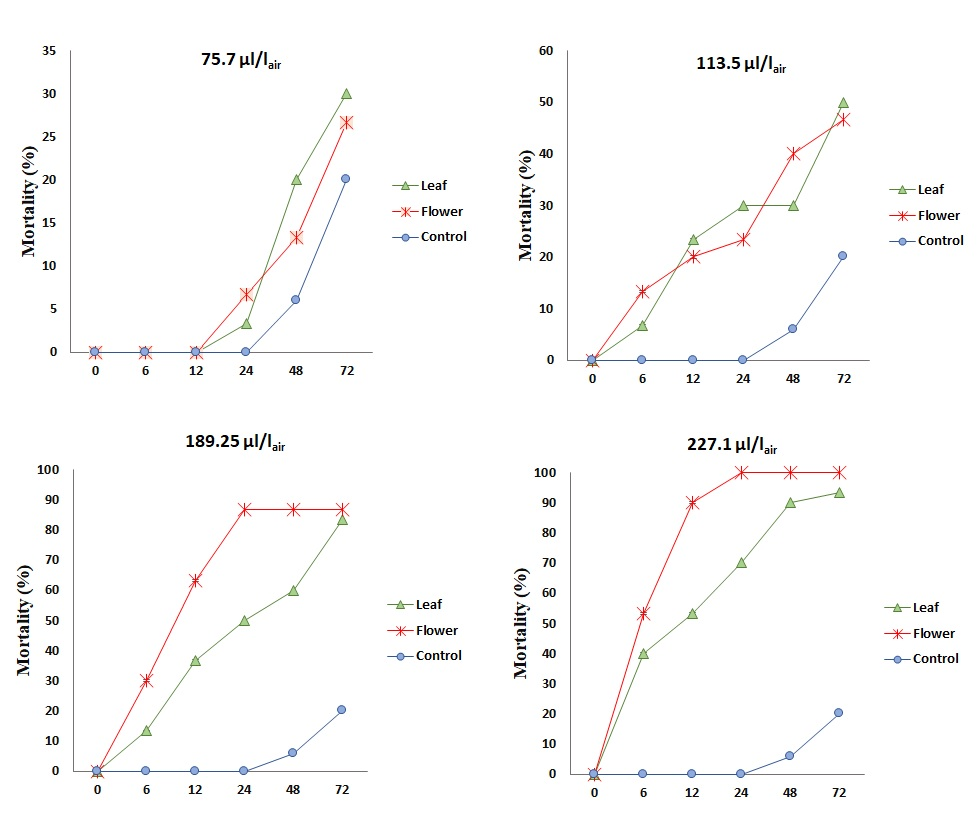


Supplementary Fig. 1. Percentage mortality of *C. maculatus* exposed for various periods (h) to different concentrations of eucalyptus leaf and flower essential oils.


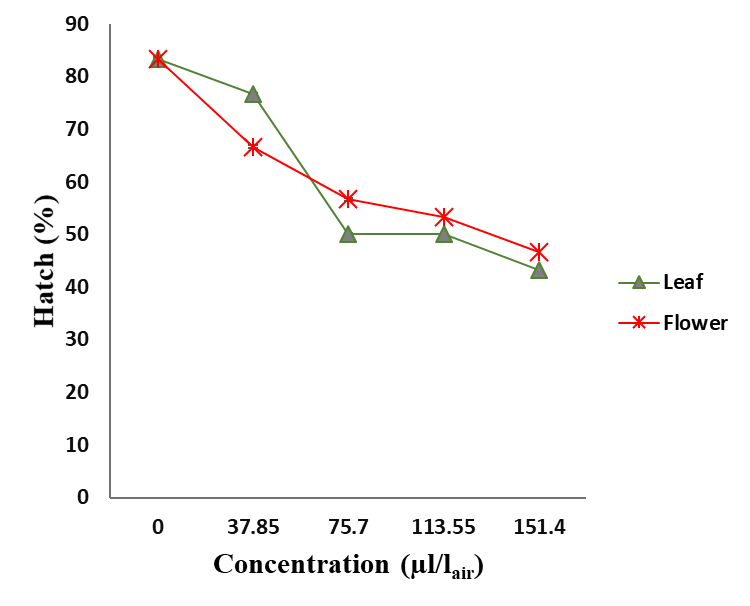


Supplementary Fig 2. Hatch rate of *C. maculatus* eggs exposed to different concentrations of eucalyptus leaf and flower essential oils.


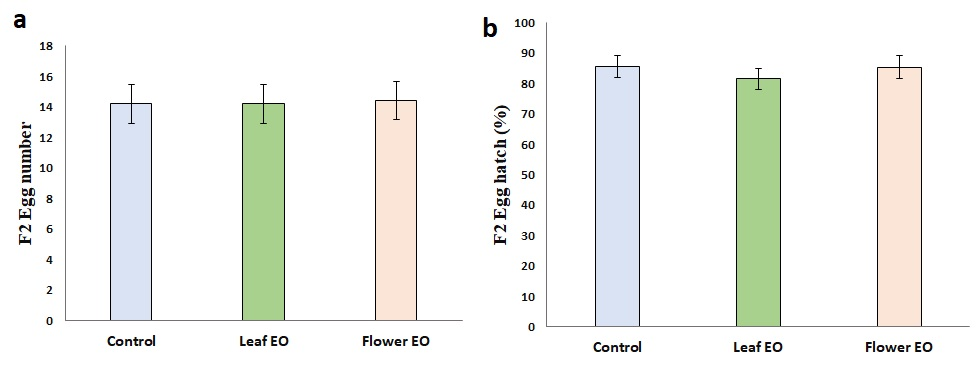


Supplementary Fig 3. Effect of eucalyptus leaf and flower essential oils on *C. maculatus* F2 egg number (a) and hath (b).
