# Supplementary material for: Callosobruchus embryo struggle to guarantee progeny production
Source: Sci Rep. 2020 Aug 6;10:13269. doi: 10.1038/s41598-020-70178-9 (PMC7413255; doi:10.1038/s41598-020-70178-9)
Supplement: Supplementary file 2 — Supplementary information 2. [file 41598_2020_70178_MOESM2_ESM.docx]

***Callosobruchus* embryo struggle to guarantee progeny production**

Azam Amiri^*1^ and Ali R. Bandani^2^

1. College of Geography and Environmental Planning. University of Sistan and Baluchestan, Zahedan, Iran.

2. Department of Plant Protection, College of Agriculture and Natural Resources, University of Tehran, Iran.

^*^Corresponding author email: azamamiri@eco.usb.ac.ir

**References**

1. Clark, M.M. & Galef, B.G. Prenatal influences on reproductive life history strategies. *Trends Ecol. Evol.* **10,** 151-153 (1995).
2. Bradley, T. J. *et al*. Episodes in insect evolution. *Integr Comp Biol*. **49,** 590-606 (2009).
3. de Roode, J. C. & Lefèvre, T. Behavioral Immunity in Insects. *Insects*. **3(3),** 789-820 (2012).
4. Gulyas, L. & Powell, J. R. Predicting the Future: Parental Progeny Investment in Response to Environmental Stress Cues. *Front Cell Dev Biol.* **7,** 115; 10.3389/fcell.2019.00115 (2019).
5. Deng, J. *et al.* Enhancement of Attraction to Sex Pheromones of *Spodoptera exigua* by Volatile Compounds Produced by Host Plants. *J Chem Ecol.* **30,**2037-2045 (2004).
6. Schoonhoven, L. M., Van Loon, J. J. A. & Dicke, M. *Insect-plant biology*. (Oxford University Press, 2005).
7. Plata-Rueda, A. *et al*. Insecticidal activity of garlic essential oil and their constituents against the mealworm beetle, *Tenebrio molitor* Linnaeus (Coleoptera: Tenebrionidae). *Sci Rep*. **7,** 46406; 10.1038/srep46406 (2017).
8. Pourya, M., Sadeghi, A., Ghobari, H., Nji Tizi Taning, C. & Smagghe, G. Bioactivity of *Pistacia atlantica* desf. Subsp. Kurdica (Zohary) Rech. F. and *Pistacia khinjuk* stocks essential oils against *Callosobruchus maculatus* (F, 1775) (Coloeptera: Bruchidae) under laboratory conditions. *J. Stored Prod. Res*. **77,** 96-105 (2018).
9. Gaire, S., Scharf, M. E. & Gondhalekar, A. D. Toxicity and neurophysiological impacts of plant essential oil components on bed bugs (Cimicidae: Hemiptera). *Sci Rep.* **9(1),** 3961; 10.1038/s41598-019-40275-5 (2019).
10. Moura, E. d. *et al*. Insecticidal activity of *Vanillosmopsis arborea* essential oil and of its major constituent α-bisabolol against *Callosobruchus maculatus* (Coleoptera: Chrysomelidae). *Sci Rep.* **9,** 3723; 10.1038/s41598-019-40148-x (2019).
11. Conchou, L. *et al*. Insect Odorscapes: From Plant Volatiles to Natural Olfactory Scenes. *Front. Physiol.* **10,** 972; 10.3389/fphys.2019.00972 (2019).
12. Vilcinskas, A. The role of epigenetics in host–parasite coevolution: lessons from the model host insects *Galleria mellonella* and *Tribolium castaneum*. *Zoology (Jena).* **119,** 273-280 (2016).
13. Knecht, A. L. *et al*. Transgenerational inheritance of neurobehavioral and physiological deficits from developmental exposure to benzo[a]pyrene in zebrafish. *Toxicol Appl Pharmacol*. **329,** 148-157 (2017).
14. Portnoy, D.S., Fields, A. T., Greer, J. B. & Schlenk, D. Genetics and Oil: Transcriptomics, Epigenetics, and Population Genomics as Tools to Understand Animal Responses to Exposure Across Different Time Scales in *Deep Oil Spills* (Murawski, S. *et al*. eds). (Springer, 2020).
15. Pavela, R. & Sedlák, P. Post-application temperature as a factor influencing the insecticidal activity of essential oil from *Thymus vulgaris*. *Ind. Crops Prod*. **113,** 46-49 (2018).
16. Viteri Jumbo, L. O., Haddi, K., Faroni, L. R. D., Heleno, F. F., Pinto, F. G., & Oliveira, E. E. Toxicity to, oviposition and population growth impairments of *Callosobruchus maculatus* exposed to clove and cinnamon essential oils. *PloS one*. **13,** e0207618 (2018).
17. Haddi, K. *et al*. Changes in the insecticide susceptibility and physiological trade-offs associated with a host change in the bean weevil *Acanthoscelides obtectus*. *J Pest Sci*. **91,** 459-468 (2018).
18. Metcalfe, N. B. & Monaghan, P. Compensation for a bad start: grow now, pay later? *Trends Ecol. Evol.* **16,** 254-260 (2001).
19. Fabian, D. & Flatt, T. Life History Evolution. Nature Education Knowledge **3(10),** 24 (2012).
20. Lü, Z. C. *et al*. Trade-offs between survival, longevity, and reproduction, and variation of survival tolerance in Mediterranean *Bemisia tabaci* after temperature stress. *J Insect Sci*. **14,** 124; 10.1093/jis/14.1.124 (2014).
21. Campolo, O., Giunti, G., Russo, A., Palmeri, V. & Zappala, L. Essential oils in stored product insect pest control. *J. Food Qual*. 1–18 (2018).
22. Alves, M. D. S. et al. Efficacy of lemongrass essential oil and citral in controlling *Callosobruchus maculatus* (Coleoptera: Chrysomelidae), a post-harvest cowpea insect pest. *Crop Prot.* **119,** 191-196 (2019).
23. Sabbour, M. M. A. Efficacy of natural oils against the biological activity on *Callosobruchus maculatus* and *Callosobruchus chinensis* (Coleoptera: Tenebrionidae). *Bull Natl Res Cent*. **43,** 206; 10.1186/s42269-019-0252-1 (2019).
24. Benelli, G. *et al*. Carlina oxide from *Carlina acaulis* root essential oil acts as a potent mosquito larvicide. *Ind. Crops Prod*. **137,** 356-366 (2019).
25. Pavela, R. *et al*. Outstanding insecticidal activity and sublethal effects of *Carlina acaulis* root essential oil on the housefly, *Musca domestica*, with insights on its toxicity on human cells. *Food Chem Toxicol.* **136,** 111037 (2020).
26. Pavela, R. Lethal and sublethal effects of thyme oil (*Thymus vulgaris* L.) on the house fly (*Musca domestica* Lin.). J*. Essent. Oil Bear. Pl*. **5,** 346-356 (2007).
27. Haddi K, Oliveira, E. E., Faroni, L. R. A., Guedes, D. C. & Miranda, N. N. S. Sublethal exposure to clove and cinnamon essential oils induces hormetic-like responses and disturbs behavioral and respiratory responses in *Sitophilus zeamais* (Coleoptera: Curculionidae). *J Econ Entomol.* **108,** 2815-2822 (2015).
28. Silva, S., Haddi, K., Viteri Jumbo, L. & Oliveira, E. Progeny of the maize weevil, *Sitophilus zeamais*, is affected by parental exposure to clove and cinnamon essential oils. *Entomol Exp Appl*. **163,** 220-228 (2017).
29. Chang, M. M. *et al*. Effect of Diallyl Trisulfide on the Reproductive Behavior of the Grain Moth, *Sitotroga cerealella* (Lepidoptera: Gelechiidae). *Insects*. **11(1),** pii: E21. 10.3390/insects11010021 (2019).
30. Papadopoulos, N. T., Katsoyannos, B. I., Kouloussis, N. A. & Hendrichs, J. Effect of orange peel substances on mating competitiveness of male *Ceratitis capitata*. *Entomol Exp Appl.* **99,** 253-261 (2001).
31. Shelly, T. E. & Mclnnis, D. O. Exposure to ginger oil enchance mating success of irradiated, mass-reared males of Mediterranean fruit fly (Diptera: Tepritidae). *J Econ Entomol*. **94,** 1413-1418 (2001).
32. Papadopoulos, N., Shelly, T., Niyazi, N. & Jang, E. Olfactory and behavioral mechanisms underlying enhanced mating competitiveness following exposure to ginger root oil and orange oil in males of the Mediterranean fruit fly, *Ceratitis capitata* (Diptera: Tephritidae). *J. Insect Behav*. 1-16 (2006).
33. Shelly, T. E. & Epsky, N. D. Exposure to Tea Tree Oil Enhances the Mating Success of Male Mediterranean Fruit Flies (Diptera: Tephritidae). *Fla. Entomol.* **98(4),** 1127-1133 (2015).
34. Morató, S., Shelly, T., Rull, J., & Aluja, M. Sexual competitiveness of *Anastrepha ludens* (Diptera: Tephritidae): Males exposed to Citrus aurantium and Citrus paradisi essential oils. *J. Econ. Entomol.***108,** 621e628 (2015).
35. Gerofotis, C. D., Ioannou, C. S. & Papadopoulos, N. T. Aromatized to Find Mates: α-Pinene Aroma Boosts the Mating Success of Adult Olive Fruit Flies. *PLoS ONE.* **8(11),** e81336; 10.1371/journal.pone.0081336 (2013).
36. den Hollander, M. & Gwynne, D.T. Female ﬁtness consequences of male harassment and copulation in seed beetles, *Callosobruchus maculatus*. *Anim. Behav.* **78,** 1061e1070 (2009).
37. Wolff, J. O. Biological functions and evolutionary aspects. In *Attachment Structures and Adhesive Secretions in Arachnids*. Biologically-Inspired Systems (ed. Gorb, S. N.) (Springer, Berlin, 2016).
38. Andrés, J. & Cordero Rivera, A. Copulation duration and fertilization success in a damselfly: an example of cryptic female choice? *Anim. Behav*. **59,** 695-703 (2000).
39. Omkar Singh, K. & Pervez, A.  Influence of mating duration on fecundity and fertility in two aphidophagous ladybirds. *J Appl Entomol.* **130,** 103–107 (2006).
40. Edvardsson, M. & Canal, D. The effects of copulation duration in the bruchid beetle *Callosobruchus maculatus*. *Behav. Ecol*. **17,** 430–434. (2006).
41. Vande Velde, L., Schtickzelle, N. & Van Dyck, H. Effect of larval food stress on male adult behavior, morphology and reproductive investment in the butterﬂy Pararge aegeria. *Evol. Ecol*. **27,** 221–234. (2013).
42. Brockmann, H. J. Alternative reproductive tactis in insects in *Alternative Reproductive Tactics: An Integrative Approach* (eds Oliveira, R. F, Taborsky, M. & Brockmann, H. J.). 177-223 (Cambridge, 2008).
43. Feil, R. & Fraga, M. Epigenetics and the environment: emerging patterns and implications. *Nat Rev Genet*. **13,** 97-109 (2012).
44. Conchou, L., Lucas, P., Meslin, C., Proffit, M., Staudt, M., & Renou, M. Insect odorscapes: from plant volatiles to natural olfactory scenes. *Front Physiol*. **10,** 972 (2019).
45. Robertson, J .L., Savin, N. E., Preisler, H. K. & Russell, R. M. *Bioassays with Arthropods*. (CRC press, 2007).
